# Influence of Optimization Design Based on Artificial Intelligence and Internet of Things on the Electrocardiogram Monitoring System

**DOI:** 10.1155/2020/8840910

**Published:** 2020-10-26

**Authors:** Ming Yin, Ru Tang, Miao Liu, Ke Han, Xiao Lv, Maolin Huang, Ping Xu, Yongdeng Hu, Baobao Ma, Yanrong Gai

**Affiliations:** ^1^The Second Medical Center and National Clinical Research Center for Geriatric Diseases, Chinese PLA General Hospital, Beijing 100853, China; ^2^Lenovo Research, Lenovo Group, Beijing 100094, China

## Abstract

With the increasing emphasis on remote electrocardiogram (ECG) monitoring, a variety of wearable remote ECG monitoring systems have been developed. However, most of these systems need improvement in terms of efficiency, stability, and accuracy. In this study, the performance of an ECG monitoring system is optimized by improving various aspects of the system. These aspects include the following: the judgment, marking, and annotation of ECG reports using artificial intelligence (AI) technology; the use of Internet of Things (IoT) to connect all the devices of the system and transmit data and information; and the use of a cloud platform for the uploading, storage, calculation, and analysis of patient data. The use of AI improves the accuracy and efficiency of ECG reports and solves the problem of the shortage and uneven distribution of high-quality medical resources. IoT technology ensures the good performance of remote ECG monitoring systems in terms of instantaneity and rapidity and, thus, guarantees the maximum utilization efficiency of high-quality medical resources. Through the optimization of remote ECG monitoring systems with AI and IoT technology, the operating efficiency, accuracy of signal detection, and system stability have been greatly improved, thereby establishing an excellent health monitoring and auxiliary diagnostic platform for medical workers and patients.

## 1. Introduction

Estimations indicate that China has 290 million cardiovascular patients, including 13 million stroke, 11 million coronary heart disease, 5 million pulmonary heart disease, 4.5 million heart failure, 2.5 million rheumatic heart disease, 2 million congenital heart disease, and 245 million hypertension patients [1]. In the recent years, the death rate from cardiovascular diseases has been the highest—greater than that from tumor and other diseases. Two out of every five deaths have been linked to cardiovascular diseases, and the death rate in rural areas has been higher than that in urban areas. In rural areas, the death rate from cardiovascular diseases is 309.33/100000, including 151.18/100000 from heart diseases. In urban areas, the death rate from cardiovascular diseases is 265.11/100000, including 138.70/100000 from heart diseases. In rural areas, the deaths from cardiovascular diseases account for 45.50% of the total deaths, whereas in urban areas, they account for 43.16% of the total deaths. Because of the large number of patients, long duration of diseases, complex etiology, high cumulative cost of treatment, and frequent doctor-patient communication, not only is the medical system under great pressure but the medical cost is also very high. Hence, it is difficult for medical resources to be used for the benefit of all sections of society [2, 3].

Current well-established electrocardiogram (ECG) monitoring systems can be mainly divided into two types. In the first type of system, only the ECG signal acquisition from the patient side is considered. The signals are directly transmitted to the doctor via GPRS or 3G/4G remote communication or transmitted to the data relay node using Bluetooth, ZigBee, or Wi-Fi; the relay node, then, sends the ECG data to the doctor through the Internet. Research on this type of system has been happening for a relatively long period, and the technology is now well established. This type of system can be the foundation for the development of other systems with similar communication architectures in the future [4–6]. However, this type of system realizes only the remote real-time recording of ECG data [7], and a doctor is still needed to perform manual ECG diagnosis [8, 9]. In the second type of system, after collecting the ECG signal from the patient, the system transmits the ECG signal to a smartphone using Bluetooth, ZigBee, or Wi-Fi, allowing the mobile phone to display the ECG waveform in real time, perform ECG analysis and diagnosis, and then, transmit the relevant information to the doctor [10–12]. However, the hardware performance of smartphones is currently limited, and they are unable to support advanced ECG diagnostic algorithms. Therefore, the ECG analysis and diagnosis results do not meet the needs of patients [13, 14]. At present, these two types of ECG monitoring systems are subject to technical limitations [15, 16]. Because of the limitations of available facilities and technologies in terms of the electronic collection, storage, and analysis of ECG data (followed by automatic diagnosis), medical service centers cannot monitor the health status of the heart in a timely and effective manner [17, 18]. Consequently, valuable opportunities for diagnosis and treatment may be missed, and hence, the needs of patients cannot be satisfied.

## 2. Optimal Design of the ECG Monitoring System

In this study, artificial intelligence (AI) is used to automate the diagnosis, annotation, and detection of ECG reports, which are accurately and effectively judged and labeled. The user's ECG signal is uploaded to the cloud in real time through the Internet of Things (IoT) and shared with the corresponding medical staff, thereby reducing the burden on the medical staff, improving the accuracy of diagnosis, and reducing human interference and the influence of human factors on the ECG report [19, 20]. By using AI, users can be monitored remotely in a timely manner, more patients can access the system, more functions can be added to the system, and answers and treatment can be provided online to specific patients [21, 22]. For patients who are not likely to visit the hospital frequently, portable ECG monitoring equipment can provide many advantages and the quality of health monitoring can be guaranteed. Doctors can collect remote real-time data, conduct health evaluations, and undertake comprehensive monitoring of relevant physiological parameters, daily living habits, and mental states of family members. In this manner, patients can receive correct and efficient treatment without leaving the home and can save treatment cost. In particular, the system can track and manage the elderly and provide medical advice and self-help training. For general hospitals, heart disease experts at different research levels can be efficiently utilized, the heavy workload of doctors can be reduced, and the diagnosis and treatment of ECG diseases can be divided into several stages (prevention, treatment, or rehabilitation) to maximize the utilization of hospital resources.

### 2.1. Optimization of System Hardware

**Figure 1:**
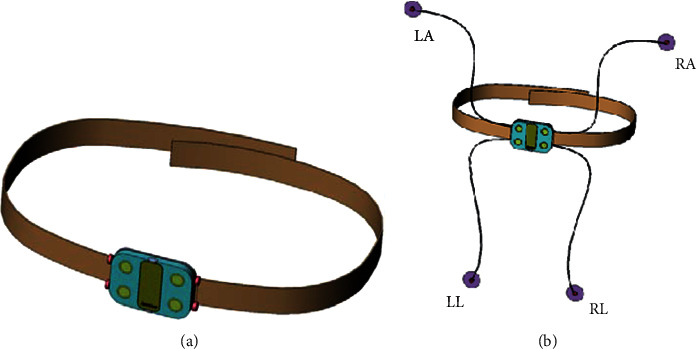
Wireless sensor equipment. LA: left arm, LL: left leg, RA: right arm, and RL: right leg.

The self-adaption and optimized wireless sensor equipment (Figure 1) can be used by residents at home. The equipment is used to detect ECG data. The wireless sensor equipment has two innovative modes, which can meet the different needs of users.

**Figure 2:**
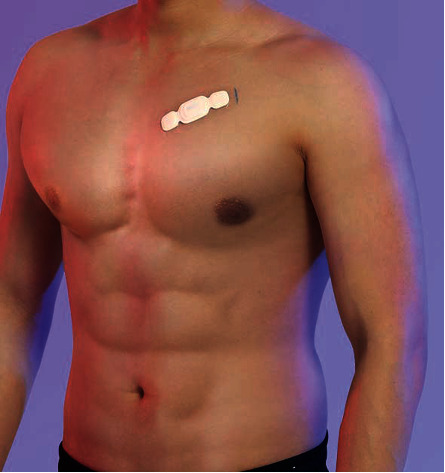
Simple low-lead method.

**Figure 3:**
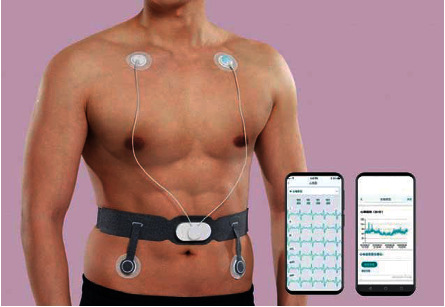
Complex multilead approach.

According to the different needs of users, different detection accuracies are required, and accordingly, different lead methods can be selected. It is recommended that users use the simple low-lead method (Figure 2) during routine examinations or when they feel healthy. The complex multilead approach (Figure 3) can be applied if the user feels sick.

The materials used so far to attach various types of sensors to the body do not meet the skin-friendly nature required for long-term wear. Certain users such as patients with acute diseases are likely to experience an episode at any time, and hence, they need to be monitored without interruption. Therefore, there is a demand to improve the probe material such that it is skin friendly and does not cause damage to the body during long-term wear while simultaneously not affecting the data collection requirements.

### 2.2. Optimization of ECG Diagnostic Algorithm of the System

Typical ECG diagnostic algorithms include three essential steps [23]: signal data preprocessing, feature extraction from data, and feature classification. Signal preprocessing is related to the ability to process information content such that features can be extracted from the content, and feature classification is closely related to the ability of represent data features. Most ECG diagnostic algorithms still have not been able to eliminate the artificial feature extraction and classification steps. Some algorithms incorporate machine learning methods based on the abovementioned three steps, and the classification ability of the algorithms is improved through feature dimension reduction and feature selection [24]. The most important aspect to be noted is that the characteristics of data are chosen subjectively. Many algorithms have been proposed before, such as the probabilistic neural network analysis method based on feature dimension reduction, support vector machine method based on feature selection, and convolution neural network based on adaptive. These diagnostic algorithms were based on the traditional algorithm and proposed that the doctor should participate in the diagnosis to offset the problem of the robustness of the algorithm caused by feature selection subjectivity and patient specificity. First, a general algorithm is trained based on a general database, and then, the first 5 min of the ECG signal of the patient is collected. Next, the collected signal is provided to the algorithm as a new sample after the doctor's diagnosis to make the algorithm obtain the specificity of the patient. Accordingly, the most ambiguous part of the patient's ECG signal, which is the most difficult to determine, is extracted and handed to the doctor for diagnosis to reduce the burden on the doctor. Although this type of algorithm effectively solves the problem of the robustness of the algorithm, it still needs the doctor's participation and cannot perform automatic ECG analysis. ECG classification algorithms are usually based on artificial features of the ECG, such as the Fourier transform and morphology [25, 26], and the wavelet analysis indicators of ECG signals. However, doctors analyze ECG signals based on their personal experience about the features to be diagnosed. Therefore, the extraction of abstract features and deep mining of the information in the signal can effectively improve the accuracy and real-time performance of the system while preventing the decrease in robustness due to feature selection subjectivity and patient specificity [27].

However, these methods still include the step of artificial feature extraction. The disadvantage of this step is that when a new sample, that is, the electroanalytical analysis information of a new patient, is provided to a typical algorithm, the robustness of the algorithm is reduced and the accuracy of the algorithm cannot be guaranteed because of feature selection subjectivity and patient specificity. Thus, misjudgment may occur.

**Figure 4:**
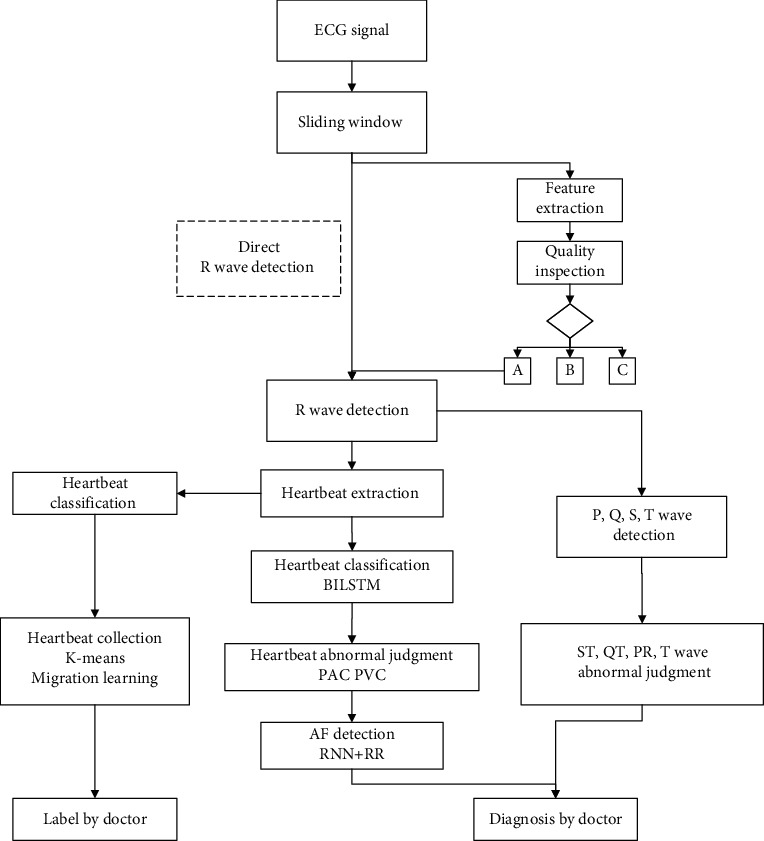
ECG algorithm process flow. Bilateral Long- and Short-Term Memory network, BiLSTM. Premature atrial complex, PAC. Premature ventricular contraction, PVC. Atrial fibrillation, AF. Recurrent neural network, RNN.

The data are uploaded to the cloud. The ECG report can be issued to the user, and the ECG data can be used for ECG trend prediction analysis. When the user's ECG signal is transmitted to the cloud, the correct signal is first identified, the filtered wave is selected, the *R* peak position is detected, the heartbeat is extracted by the *R* peak, and the heartbeat is classified by the Bidirectional Long- and Short-Term Memory network (Bilateral Long- and Short-Term Memory network, BiLSTM) method. Then, the algorithm detects ECG abnormalities, including Premature Ventricular Contraction (PVC), Premature Atrial Complex (PAC), and Atrial Fibrillation (AF). Finally, the labeled heartbeat data are used to train various classification algorithms such as neural networks, support vector machines, and logistic regression. The neural network with the best performance is selected as the ECG algorithm classifier, which completes the study of the ECG algorithm. According to the report, the corresponding doctor will be asked to treat the patient. The entire ECG algorithm process flow is shown in Figure 4.

**Figure 5:**
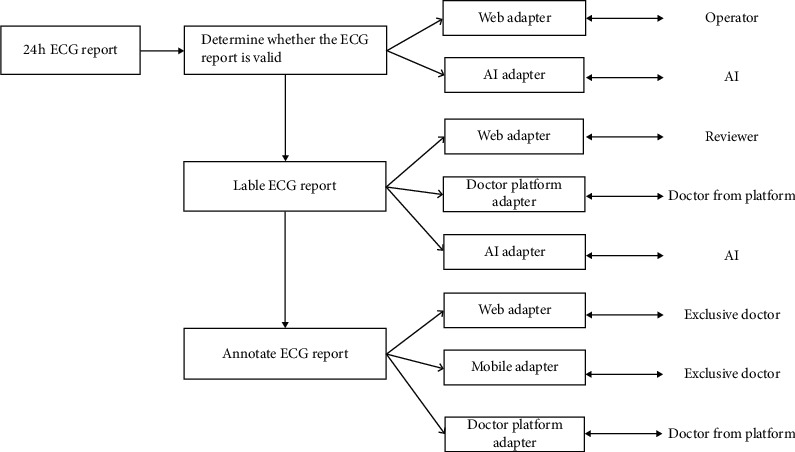
24 h ECG report grading delivery mechanism.

After the automatic monitoring of the ECG signal is performed, the report is processed and distributed by the AI method, and the ECG report is dispatched, which increases the accuracy and timeliness of the report distribution while simultaneously reducing the burden of the operator, planner, and doctor. Furthermore, the AI workload will be gradually increased in the future to reduce manual operations. The 24 h ECG report grading delivery mechanism is shown in Figure 5. Finally, AI is expected to completely replace manual operations.

## 3. Automatic ECG Algorithm Test

In this paper, fast Convolutional Neural Networks (fast-CNNs) algorithm is used to process the one-dimensional ECG signal in two-dimensional graphics, so that the signal can be comprehensively grasped from a higher dimension. The algorithm uses a 32-layer convolution network structure to extract different levels of features from the input ECG graphic signals and can obtain useful features from the whole and subdivision levels. To evaluate the accuracy of the ECG algorithm and its various functional modules, two algorithm evaluation tests are performed. The first is based on standard ECG databases such as MIT-BIH (Table 1). By comparing the labeled information of each heartbeat, according to the YY 0885-2013 standard analysis algorithm, the sensitivity and true positive details are detected with respect to QRS, Ventricular Ectopic Beat (VEB), and Supraventricular Ectopic Beat (SVEB).

Next, data are collected from a real human body using a dynamic wearable ECG device and verified using the doctor's Lenovo-SEU-DB dataset, and the accuracies of different methods in each functional module are compared and evaluated. The details are shown in Table 2.

Although the classification accuracy of ECG algorithm in this paper may not be the best, it does not need to extract the complex signal features manually, and the algorithm itself can extract the ECG signal. Features are classified and recognized to achieve acceptable classification accuracy. Even if some cases are obviously disturbed by noise, the classification accuracy is acceptable.

Through the abovementioned two examples, we can see that the fast-CNN algorithm used in this paper can guarantee the high accuracy of the CNN for QRS wave detection and the real-time detection. This algorithm has the following advantages:

1. After using this algorithm, it has higher detection sensitivity and accuracy for QRS detection and has better adaptability for worse Signal Noise Ratio (SNR) level: the algorithm adopts a 32-layer convolution network structure. In this way, compared with the traditional ECG detection algorithm, it has stronger robustness and adaptability to noise signals and higher detection accuracy.
2. Although some existing algorithms, such as Regions with CNN features (R-CNN) and sppnet, make the deep neural network have some new technical breakthroughs in the field of target detection in ECG, it is far from the true real-time detection and end-to-end results. In this paper, fast-CNN is applied to the one-dimensional signal of ECG from the creative graph detection to ensure the real-time detection.

## 4. Conclusions

Through the requirements of remote ECG monitoring, a set of remote ECG monitoring schemes is put forward, and the requirements analysis and system design, the design of remote ECG monitoring system, and the design of ECG diagnosis algorithm are carried out. Finally, the remote ECG monitoring system is tested.

The main work of this paper is as follows:

1. A three-layer structure of “acquisition end server end user end” of the remote ECG monitoring system is proposed, and a set of hardware platforms with signal acquisition and transmission function is built by using the existing hardware equipment to realize the acquisition and upload function of ECG signal
2. The collection end realizes the collection and upload function of the ECG signal, the server end realizes the storage management of data, the execution of diagnosis algorithm, and the response to the request of the user end, and the user end realizes the functions of user interface design, ECG drawing, and signal data acquisition. According to the principle of compatibility and expansibility, the software development platform of the system is built, which lays a solid foundation for the follow-up development and research.
3. The ECG diagnosis algorithm and system of remote ECG monitoring system are tested. The performance of ECG diagnosis algorithm is tested and compared with the latest algorithm in feature engineering design and classification accuracy. The function of the system is tested, and the functions of the system are tested from the perspective of users. The test results show that each module of the remote monitoring system works normally and has a certain accuracy rate of arrhythmia diagnosis, which meets the expected requirements.
4. Signal feature extraction needs further optimization. In the follow-up study, a variety of different network layers can be used for testing to achieve the best feature extraction effect.

## Figures and Tables

**Table 1:** Algorithm test results based on MIT-BIH ECG databases

Database	Record	Total beats	QRS Se (%)	QRS P+ (%)
mitdb	100	2273	99.78	100.00
mitdb	101	1865	99.89	99.89
mitdb	102	2187	98.63	100.00
mitdb	103	2084	99.62	100.00
mitdb	104	2229	97.76	99.41
mitdb	105	2572	99.92	99.65
mitdb	106	2027	95.56	100.00
mitdb	107	2137	99.58	100.00
mitdb	108	1763	99.43	99.72
mitdb	109	2532	99.64	100.00
mitdb	111	2124	99.72	100.00
mitdb	112	2539	99.80	100.00
mitdb	113	1795	99.78	100.00
mitdb	114	1879	99.73	100.00
mitdb	115	1953	99.80	100.00
mitdb	116	2412	98.92	100.00
mitdb	117	1535	99.80	100.00
mitdb	118	2278	99.87	100.00
mitdb	119	1987	90.59	100.00
mitdb	121	1863	99.73	100.00
mitdb	122	2476	99.80	100.00
mitdb	123	1518	99.60	100.00
mitdb	124	1619	98.95	100.00
mitdb	200	2601	99.58	100.00
mitdb	201	1963	95.67	100.00
mitdb	202	2136	98.92	100.00
mitdb	203	2980	96.31	100.00
mitdb	205	2656	99.62	100.00
mitdb	207	2332	87.61	100.00
mitdb	208	2955	75.84	100.00
mitdb	209	3005	99.83	100.00
mitdb	210	2650	97.09	100.00
mitdb	212	2748	99.82	100.00
mitdb	213	3251	98.83	100.00
mitdb	214	2262	99.69	100.00
mitdb	215	3363	99.58	100.00
mitdb	217	2208	99.46	100.00
mitdb	219	2154	99.49	100.00
mitdb	220	2048	99.76	100.00
mitdb	221	2427	96.79	100.00
mitdb	222	2483	98.23	100.00
mitdb	223	2605	94.89	100.00
mitdb	228	2053	95.91	100.00
mitdb	230	2256	99.78	100.00
mitdb	231	1571	99.81	100.00
mitdb	232	1780	99.89	99.94
mitdb	233	3079	99.32	100.00
mitdb	234	2753	99.71	100.00
		Average	98.07	99.97

**Table 2:** Accuracy of different methods in each functional module.

Index	Fast-CNN				QRS based by P&T			
	Se	PPV	Acc	F1	Se	PPV	Acc	F1
1	0.9953	0.9908	0.9863	0.9931	0.9958	0.9922	0.9881	0.994
2	0.9716	0.9941	0.966	0.9845	0.9857	0.9834	0.9695	0.9827
3	0.9752	0.9857	0.9616	0.9804	0.9079	0.9128	0.8354	0.9103
4	0.9953	0.9995	0.9948	0.9974	0.9995	0.9991	0.9986	0.9993
5	0.986	0.9754	0.9621	0.9807	0.9808	0.9586	0.941	0.9696
6	0.9645	0.9828	0.9484	0.9735	0.9774	0.9738	0.9524	0.9756
7	0.9919	0.986	0.9781	0.9889	0.9953	0.979	0.9745	0.9871
8	0.9881	0.9851	0.9736	0.9866	0.9902	0.9868	0.9772	0.9885
9	0.9827	0.9596	0.9437	0.971	0.9637	0.9529	0.9199	0.9583
10	0.9592	0.9929	0.9526	0.9895	0.9865	0.9924	0.9791	0.9757
11	0.9939	0.9747	0.9688	0.9842	0.9898	0.9099	0.9015	0.9482
12	0.9978	0.9974	0.9952	0.9976	0.9958	0.9908	0.9866	0.9933
13	0.991	0.9959	0.9869	0.9934	0.9943	0.9762	0.9708	0.9852
14	0.983	0.9862	0.9697	0.9846	0.9898	0.9728	0.9631	0.9812
15	0.9796	0.9572	0.9384	0.9682	0.985	0.9806	0.9662	0.9828
16	0.9402	0.9461	0.8924	0.974	0.9749	0.9731	0.9493	0.9432
17	0.9844	0.9818	0.9668	0.9831	0.9757	0.9636	0.941	0.9696
18	0.9489	0.9644	0.9168	0.9566	0.9758	0.9634	0.9409	0.9696
19	0.9815	0.9923	0.974	0.9869	0.9834	0.9814	0.9654	0.9824
20	0.9811	0.9907	0.9721	0.9858	0.9814	0.9816	0.9637	0.9815
AVR	0.9796	0.9819	0.9624	0.9807	0.9814	0.971	0.9542	0.9762

Convolutional Neural Network, CNN. Sensitivity, Se. Positive predictive value, PPV. Accuracy, Acc. F1- measure, change the value of F function by adjusting alpha, F1 when alpha = 1.

## Data Availability

The data used to support the findings of this study are available from the corresponding author upon request.
